# Supplementary material for: Knowledge and practice of clients on preventive measures of COVID-19 pandemic among governmental health facilities in South Wollo, Ethiopia: A facility-based cross-sectional study
Source: PLoS One. 2021 Mar 1;16(3):e0247639. doi: 10.1371/journal.pone.0247639 (PMC7920351; doi:10.1371/journal.pone.0247639)
Supplement: Attachment — Submitted filename: Response to reviewer.docx [file pone.0247639.s002.docx]

Response to reviewer	Date: 16 December/2020

PONE-D-20-23137

**Knowledge and Practices of Clients on preventive measures of COVID-19 Pandemic among Governmental health facilities in South Wollo, Ethiopia: A Facility-based Cross-Sectional study**

**Erkihun Tadesse**

**PLOS ONE**

Dear all,

We would like to thanks for these constructive, building and improvable comments on this manuscript that would improve substance and content of the manuscript. We considered each comments and clarification questions of editors and reviewers on the manuscript thoroughly. Our point-by-point responses for each comment and questions are described in detailed on the following pages. Further, the details of changes were shown by highlighting in the revised manuscript file attached.

**Editor comments**

1. Please ensure that your manuscript meets PLOS ONE's style requirements, including those for file naming. The PLOS ONE style templates can be found:

<https://journals.plos.org/plosone/s/file?id=wjVg/PLOSOne_formatting_sample_main_body.pdf>  and

[https://journals.plos.org/plosone/s/file?id=ba62/PLOSOne_formatting_sample_title_authors affiliations.pdf](https://journals.plos.org/plosone/s/file?id=ba62/PLOSOne_formatting_sample_title_authors%20affiliations.pdf)

**Response**: Thank you editor for the supportive comment. Our manuscript meets PLOS ONE's style requirements.

2. We suggest you thoroughly copyedit your manuscript for language usage, spelling, and grammar.

**Response**: Thank you very much editor for the supportive suggestions to improve our work for readability. We amended the language, spelling and grammar in the revised manuscript file shown by highlighting.

3. Your ethics statement should only appear in the Methods section of your manuscript. If your ethics statement is written in any section besides the Methods, please move it to the Methods section and delete it from any other section. Please ensure that your ethics statement is included in your manuscript, as the ethics statement entered into the online submission form will not be published alongside your manuscript.

**Response**: Thank you very much editor for the supportive comments to improve our work. We take the ethics statement to the methods section and deleted it from the declaration section in the revised manuscript file shown by highlighting.

4. Please include a separate caption for each figure in your manuscript.

**Response**: Thank you very much editor for the supportive comment and we included caption for figure 1 and figure 2 in the manuscript file as shown by highlighting.

**Reviewer 1 comments**

1. The sampling methods not clear in relation to facility selection and randomization. Number of sample is relatively low to be representing. Use of old census for the catchment area for sample calculation without estimation of expected growth affects the result generalization. Selection bias due to facility base sampling does not support community base result.

**Response**: Thank you reviewer for the constructive and supportive comments to improve our work for readability. We clarified the sampling methods in relation to sample size determination, facility selection and randomization in the revised manuscript file from line number **115-129**.

-“We calculated the sample size using **score test for one sample proportion** by STATA version 14 with the assumption that 50% of clients had good knowledge that scored above the mean, 5% level of significance (α), power 80% (β), and at least 16% of client’s knowledge improvement is expected after six months of the pandemic duration (December to May). Thus, the total calculated sample size was 83 clients.”

-“We selected Dessie town purposively from south wollo zone since it is hotspot for the spread of the virus as speculated by the Ethiopian Public Health Research Institute and selected by the federal ministry of health of Ethiopia as the only treatment and screening center for COVID-19 during the early phase of the pandemic in the country. We selected 3 health facilities namely Dessie Referral hospital, Segno Gebeya health center, and Buanbuawuha health centers randomly using lottery method. Then, we proportionally allocated the sample size to each health facility based on the patient flow of the facilities. Finally, we selected 43 participants from Dessie referral hospital, 20 participants from Segnogebeya health center, and 20 participants from Buanbuawuha health center consecutively.”

As a limitation of this study we considered small sample size in which the finding from the study may not be representative/ not enough for generalization. Since we conducted this study during the early phase of the pandemic in the country, Ethiopia where there is no evidence (study) about the level of knowledge and preventive practice of the COVID-19 pandemic in the country including our study area. Thus, we conducted this study within 9 days of data using 81 participants attending the selected health facilities to provide urgent evidence (feedback) to the concerned bodies to halt further progression of the pandemic by designing appropriate prevention and control strategies.

**Reviewer 2 comments**

-Statistical part written good, used statistical tests described, approved with the tables and graphs. There is some concern about sample size (81) and duration of the study. Is it enough for generalizing the result with that sample size, and why the duration only 9 days ???

For that reason the question: Has the statistical analysis been performed appropriately and rigorously? - answered don’t know. The article itself well written, English in submitted article is clear, understandable, article style used.

**Response**: Thank you reviewer for the constructive and supportive comments to improve our work for readability. As a limitation of this study we considered small sample size in which the finding from the study may not be representative/ not enough for generalization and described in the discussion section of the manuscript file.

We conducted this study during the early phase of the pandemic in the country, Ethiopia where there is no evidence (study) about the level of knowledge and preventive practice of the COVID-19 pandemic in the country including our study area. In spite of this limitation, we conducted this study within 9 days of data using 81 participants attending the selected health facilities to provide urgent evidence (feedback) to the concerned bodies to halt further progression of the pandemic by designing appropriate prevention and control strategies.
